# 1-Kestose, the Smallest Fructooligosaccharide Component, Which Efficiently Stimulates *Faecalibacterium prausnitzii* as Well as Bifidobacteria in Humans

**DOI:** 10.3390/foods7090140

**Published:** 2018-09-01

**Authors:** Takumi Tochio, Yoshihiro Kadota, Toshio Tanaka, Yasuhiro Koga

**Affiliations:** 1 Research and Development Center, B Food Science Co., Ltd., Aichi 478-0046, Japan; t-tochio@bfsci.co.jp (T.T.); y-kadota@bfsci.co.jp (Y.K.); 2 Department of Cardiology, Osaka Habikino Medical Center, Osaka 583-8588, Japan; ttanak@ra.opho.jp; 3 Department of Gastroenterology, Tokai University School of Medicine, Isehara 259-1193, Japan; 4 Japanese Society for Probiotic Science, Isehara 259-1143, Japan

**Keywords:** prebiotics, 1-kestose, fructooligosaccharides, *Faecalibacterium prausnitzii*, butyrate

## Abstract

The concept of prebiotics was established more than 30 years ago. While the prebiotic concept has now expanded thus includes non-carbohydrate substances and diverse categories other than foods, fructooligosaccharides (FOS) have still predominantly been used as pebiotics, because the effects of FOS exclusively act through the enrichment of *Bifidobacterium* and *Lactobacillus* spp., which have been classified as beneficial intestinal commensals so far. Now the commercially available FOS products are synthetic mixture of several kinds of FOS components including 1-kestose (GF_2_), nystose (GF_3_) and GF_4_. In our previous studies, superiority of 1-kestose to the longer-chain FOS components such as nystose with regard to bifidogenic activity was clearly demonstrated. Recently, a broader range of beneficial bacteria including butyrate-producing indigenous bacteria have been recognized and expected to be new probiotic strains. Among them, resident *Faecalibacterium prausnitzii* is a butyrate producer with a significant anti-inflammatory effect thus expected to be useful as a next-generation probiotic. However, this bacterium is extremely oxygen-sensitive thus can be difficult to grow industrially. On the other hand, we have clearly demonstrated a significant prebiotic effect of 1-kestose, which is the smallest component of FOS, on *F. prausnitzii* in the gut of humans. These findings suggest that 1-kestose has impressive potential as a new prebiotic targeting *F. prausnitzii*, a next-generation probiotic strain, as well as bifidobacteria.

## 1. Fructooligosaccharides Have Been the Major Prebiotics

In 1985, Gibson et al. [1] defined a prebiotic as a non-digestive food ingredient that beneficially affects the host by selectively stimulating the growth and/or activity of the beneficial bacteria in the intestine, thereby improving host health. Thus, in order to be classified as a prebiotic, the compound should not be digested or absorbed in the upper part of the gastrointestinal tract before reaching the lower part of intestine. In addition, it also should be a selective substrate for indigenous bacteria in the intestine, which are beneficial to the host. Since this initial description, a number of milestones in prebiotic research have been achieved using non-digestible carbohydrates and poly- or oligo-saccharides. The most widely used of these compounds are fructans, including both relatively short-chain fructooligosaccharides (FOS; degree of polymerization (DOP 2–9) and long chain inulin (DOP 10–60). Recently, the International Scientific Association for Probiotics and Prebiotics (ISAPP) expanded the concept of prebiotics. According to the ISAPP consensus statement [2], the concept of prebiotics also includes non-carbohydrate substances, applications to body sites other than the gastrointestinal tract, and diverse categories other than foods, while the original definition of the prebiotic remains unchanged.

Bifidobacteria and lactobacilli can be classified as beneficial intestinal commensals. The genus *Bifidobacterium* is a major group of glycolytic bacteria in the large intestine of humans, comprising up to 25% of the total population in the gut of adults and more than 90% in infants [3]. As mentioned above, FOS and inulin have predominantly been used so far because they were shown to stimulate the growth of indigenous bifidobacteria, which, after several days of the treatment become predominant in the intestine [3].

**Figure 1:**
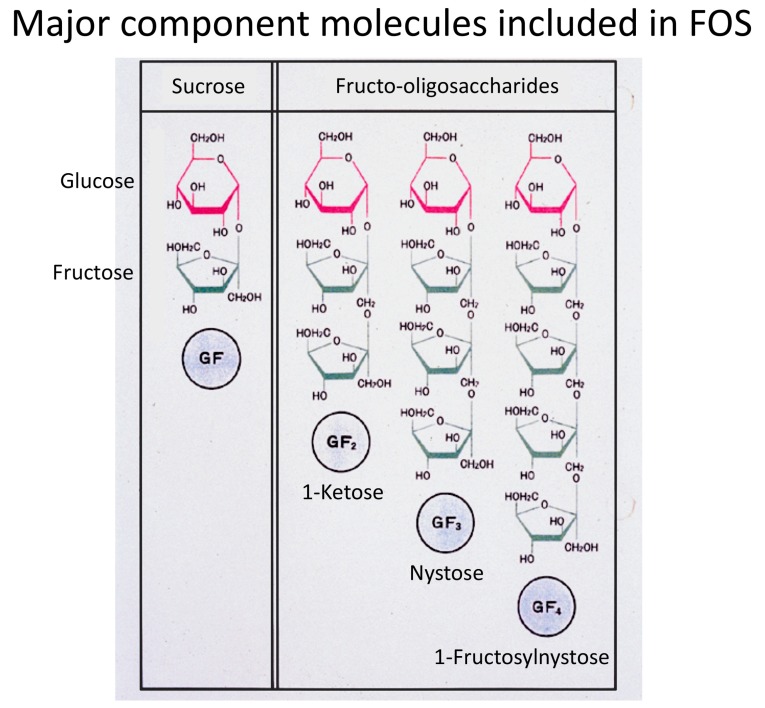
Structures of the shortest (GF_2_: 1-kestose), second shortest (GF_3_: nystose), and third shortest (GF_3_: 1-fructosylnystose) components of fructooligosaccharides (FOS) are shown.

While FOS exist widely in plants such as onion, asparagus root, and tubers of Jerusalem artichoke, commercially available FOS are produced from sucrose with the aid of β-fructofuranosidase. For example, Meioligo^®^ (Meiji Co., Ltd., Tokyo, Japan) is a representative FOS product made in Japan. This product is a synthetic mixture of several kinds of FOS components, consisting of 36% 1-kestose (glucose-fructose-fructose, GF_2_), 51% nystose (GF_3_), and 9% 1-β-fructofuranosyl-nystose (GF_4_) (Figure 1). However, which FOS component in the mixture of FOS components is really involved in the selective stimulation of bifidobacteria has been unclear. Therefore, we examined the difference in the stimulating activity between 1-kestose and nystose, both of which are dominant components in synthetic FOS products, using various kinds of bifidobacteria strains as the targets in the culture system as described below [4].

## 2. Superiority of 1-Kestose to the Longer-Chain FOS Components Such as Nystose with Regard to Bifidogenic Activity

**Figure 2:**
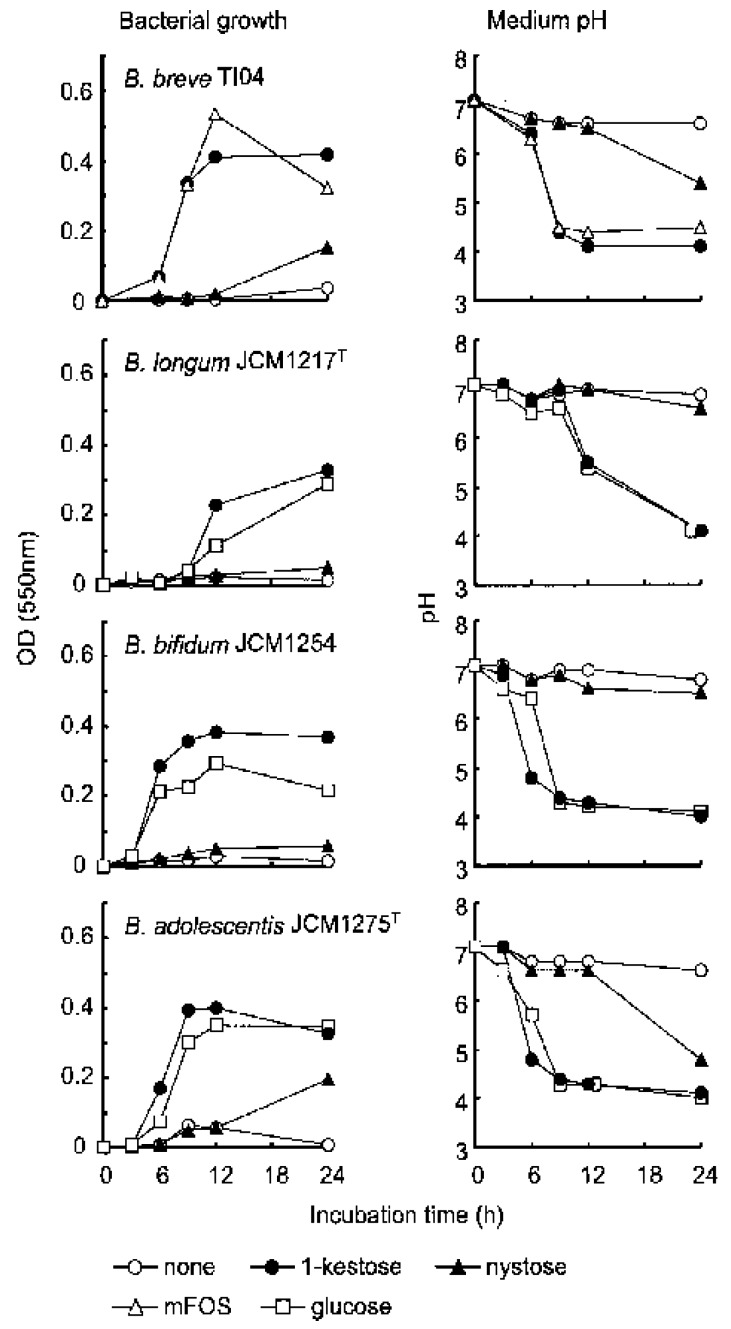
Growth of bifidobacteria in the culture together with FOS. Each *Bifidobacterium* strain was suspended in broth at 10^7^ CFU/mL together with 1.0% (*w*/*v*) of each component of FOS, a mixture of FOS, or glucose and then incubated for 24 h in triplicate. Broth with neither FOS nor glucose (none) is also shown. At 0, 3, 6, 9, 12, and 24 h after the start of incubation, aliquots were taken to measure the OD and pH values. Each plot represents the mean of three samples. (Quoted from [4] with permission).

In our previous study [4], four kinds of *Bifidobacterium* species including *B. breve, B. longum, B. bifidum,* and *B. adolescentis* were used as the target bifidobacteria in the culture to which 1-kestose (supplied from B Food Science Co., Ltd., Tokyo, Japan) nystose (B Food Science Co., Ltd., Tokyo, Japan), mFOS (mixture of FOS components: Meioligo^®^), or glucose (control) was added (Figure 2). As a result, the bacterial growth of all of the *Bifidobacterium* species was far more rapid and greater in the culture to which 1-kestose was added than in the culture to which nystose was added. (Figure 2, the left panels). The decrease in the medium pH, which means an increase in the metabolic activity of the bacteria, was compatible with the growth of the bacteria. That is, the pH value decreased much in the cultures to which 1-kestose was added, but only a little in the cultures to which nystose was added (Figure 2, the right panels).

The deficiency in the bifidogenic effect by nystose was marked when *B. longum* and *B. bifidum* were used as the targets with neither significant bacterial growth nor a pH decrease found in the cultures to which nystose was added (Figure 2). Therefore, while mFOS significantly stimulated *B. breve* in the culture, it is likely that the bifidogenic effect was exerted not by nystose but by the 1-kestose included in mFOS. Given that the major component of many of synthetic FOS products such as Meioligo^®^ is nystose, the effective use of those FOS products as a prebiotic appears dubious from a pharmacological viewpoint. Given that one of the cardinal criteria of prebiotics is the selective utilization by beneficial bacteria, it may be inappropriate to use a mixture of FOS products as the best prebiotic.

**Figure 3:**
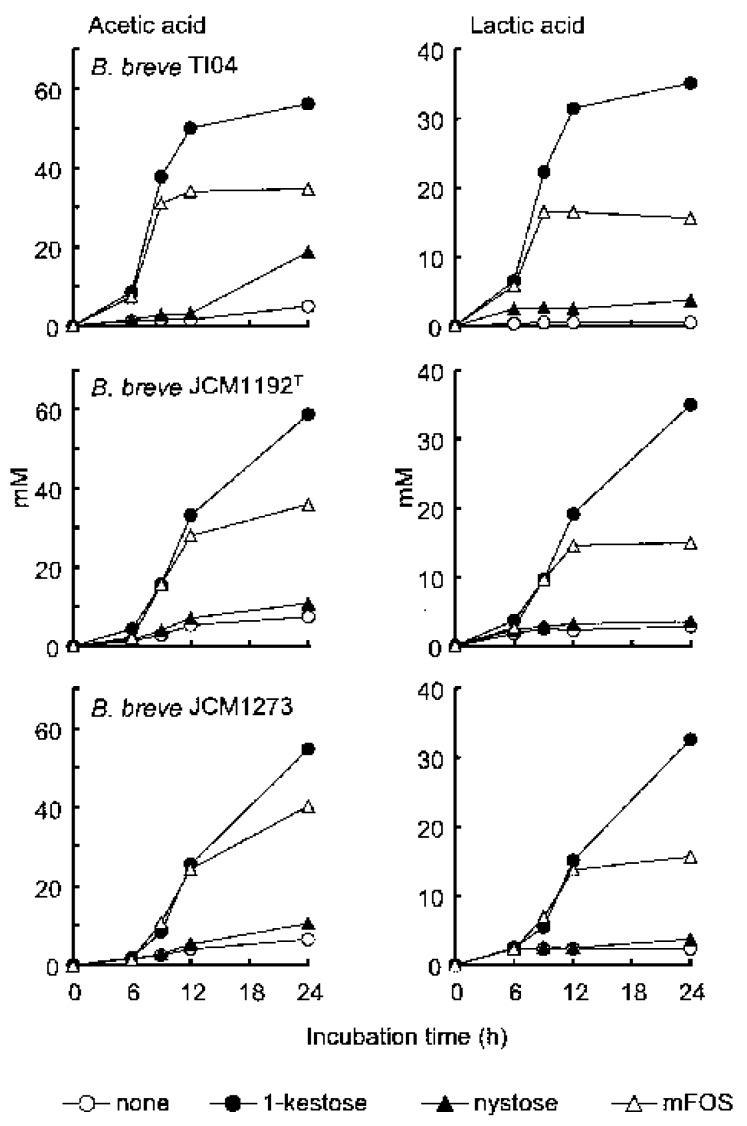
Amount of organic acids in the culture supernatant. Each *B. breve* strain was suspended in broth at 10^7^ CFU/mL together with 1.0 % (*w*/*v*) of each component of FOS or a mixture of FOS and then incubated for 24 h in triplicate. The culture without any FOS (none) is also shown. At 0, 3, 6, 9, 12, and 24 h after the start of incubation, aliquots of the supernatants were taken to measure the concentration of both acetic and lactic acids. Each plot represents the mean of three samples. (Quoted from [4] with permission).

To further ascertain that bifidobacteria preferentially ferment 1-kestose and minimally ferment nystose in FOS, the amount of short-chain fatty acids (SCFAs) and lactic acid produced in the bifidobacteria culture was measured using three kinds of *B. breve* strains, as these organic acids were the major products of prebiotic metabolism by bifidobacteria (Figure 3). As expected, a large production of SCFA (acetate) was found in all of the *B. breve* strains cultures to which 1-kestose was added, but only marginal production was observed in the cultures to which nystose was added. Taken together, these findings underscore the remarkable superiority of 1-kestose to nystose with regard to the bifidogenic activity.

Such marked differences in the utilization and resultant bifidogenic activity between 1-kestose and nystose were suggested to be due to the difference in the number of DOP (degree of polymerization) as the numbers in 1-kestose and nystose are 2 and 3, respectively. A lower number of DOP in shorter-chain FOS is thought to be associated with higher utilization. In the investigation into the utility of FOS and inulin using 55 different *Bifidobacterium* strains, Rossi et al. [5] reported that the FOS, which have a DOP of 2 to 9, were easily utilized by most strains, whereas inulin, which has a DOP of 10 to 60, was used by only 8 of 55 strains. Furthermore, in their continuous culture study incubating bifidobacteia together with a mixture of FOS components with various chain lengths, the shorter-chain FOS components were consumed first, whereas the longer chain FOS components disappeared more slowly, supporting the idea that the number of DOP in FOS is a critical determinant for bifidobacteria to facilitate fermentation. Several reports were also in agreement with this idea that bifidobacteria prefer short-chain galactooligosaccharides (GOS) as well as short-chain FOS as a substrate for growth [6,7]. While the mechanism still needs to be clarified, it is suggested that the shorter-chain FOS are easier for the transport machinery on the bifidobacteria to capture and deliver into the bacterial cytoplasm for fermentation [8].

The ingestion of a high amount of oligosaccharides often cause unpleasant symptoms such as diarrhea, abdominal distention, and flatulence probably due to non-selective stimulation of non-beneficial bacteria and/or remaining without utilization in the intestine. Therefore relatively ineffective fructans with regard to bifidogenic effect, such as longer-chain FOS and inulin, might be removed from prebiotic FOS products to the greatest degree possible. Instead, purified 1-kestose should be used in light of ability to induce an efficient prebiotic effect at a low amount.

## 3. *Faecalibacterium prausnitzii* as a Next-Generation Probiotic Capable of Producing Butyrate

*F. prausnitzii* is a non-spore-forming and non-motile Gram-positive bacterium belonging to the Class *Clostridium*. The members of *Clostridium* cluster IV (the *Clostridium leptum* group) are closely related to *F. prausnitzii* in terms of butyrate production. *F. prausnitzii* accounts for 5% to 15% of total intestinal microbiota in healthy adults and is considered one of the most-abundant butyrate-producing bacteria alongside *Clostridium* clusters IV and XIVa in the human intestinal tract [9].

Accumulating evidence has shown that the relative abundance of *F. prausnitzii* can serve as an indicator or biomarker of intestinal health in adults. It was reported that a reduction in the number of this bacterium in the intestine is associated with an increased risk of postoperative recurrence of ileal Crohn’s disease [10]. A lower proportion of this bacterium in resected ileal Crohn mucosa was also associated with endoscopically diagnosed recurrence. In addition, *F. prausnitzii* has been revealed to exert anti-inflammatory effects [11]. In vitro peripheral blood mononuclear cell stimulation by this bacterium led to significantly lower production of pro-inflammatory cytokines, such as IL-12 and IFN-γ, but much higher secretion of anti-inflammatory cytokines like IL-10 [10]. Possible mechanisms of the anti-inflammatory effect exerted by *F. prausnitzii* are as follows; (1) factors produced during anaerobic growth by this bacterium inhibit NF-κB activation, thereby suppressing the generation of pro-inflammatory cytokines in the host cells [10]; (2) *F. prausnitzii* has capacity to induce a relatively large amount of IL-10 in peripheral blood mononuclear cells and dendritic cells, which subsequently induce the production of Foxp3^+^ Tregs and enhance their suppressive activity in the intestinal mucosa [12]; and (3) butyrate produced by this bacterium induces the differentiation of colonic Tregs [13].

## 4. Biological Effects of Butyrate Secreted by Butyrate-Producing Bacteria Including *F. prausnitzii* in the Intestine

Among SCFAs, butyrate exerts a dominant effect on colonic health [14]. The functions of butyrate in promoting colonic health range from being an energy source for intestinal epithelial cells to being a critical mediator of anti-inflammatory effects as briefly mentioned in the previous paragraph. Of note, butyrate is never produced by bifidobacteria or lactobacilli although acetate is produced in large amounts [2]. While acetate secreted by bifidobacteria is changed into butyrate by cross-feeding bacteria in the gut microbiota, the composition of those surrounding bacteria determine how much butyrate is ultimately produced in the gut when exclusively bifidogenic prebiotics are administered.

A study of Gpr109a, a receptor for both butyrate and niacin (vitamin B3), clearly demonstrated the biological roles of the butyrate secreted by gut microbiota in the intestine using the Gpr109a gene knock-out mice [15]. In that study, the Gpr109a receptor was expressed in both hematopoietic and non-hematopoietic cells. The analysis revealed that Gpr109a signaling promoted anti-inflammatory properties in colonic macrophages and dendritic cells, enabling these immune cells to induce the differentiation of Treg cells and IL-10-producing T cells.

## 5. Difficulty Using *F. prausnitzii* as a Probiotic

As mentioned above, *F. prausnitzii* is a butyrate producer with a significant anti-inflammatory effect. This bacterium has therefore been expected to be useful as a next-generation probiotic, especially for the treatment of inflammatory diseases. However, *F. prausnitzii* is an extremely oxygen-sensitive bacterium and thus can be difficult to grow industrially [16]. Indeed, Wrzoseck et al. [17] reported that they were unable to obtain *F. prausnitzii*-mono-associated gnotobiotic rats because the oxidoreduction potential in the intestine of germ-free rats was still too high for this bacterium to colonize there. Only after reducing the oxidoreduction potential after implanting other anaerobic bacteria in the intestine, *F. prausnitzii* was able to colonize the intestine. Given the difficulty in the mass-growing probiotic *F. prausnitzii*, the stimulation of resident *F. prausnitzii* using prebiotics may be an alternative method of capitalizing on the beneficial effects of this bacterium for human health.

## 6. Evidence of the Prebiotic Effects of 1-Kestose on Both Atopic Dermatitis and Beneficial Bacteria

### 6.1. Evidence of the Prebiotic Effect of 1-Kestose on Beneficial Bacteria Other Than Bifidobacteria in Humans

Because bifidobacteria have been presumed to exert a beneficial effect on atopic dermatitis (AD), several attempts have been made to administer prebiotic oligosaccharides in order to increase and activate indigenous bifidobacteria in the AD patients [18]. In a small-scale randomized controlled trial, we also assessed the clinical effect of 1-kestose on infants with atopic dermatitis (AD), as 1-kestose has been shown to be the most efficient FOS component for stimulating bifidobacteria [19]. While the symptoms of AD were significantly improved in the patients treated with 1-kestose, no significant correlation was found between the degree of symptom improvement and the increase in the fecal bifidobacteria count. These results suggest that some bacteria other than bifidobacteria might be involved in the improvement of clinical symptoms in AD patients treated with 1-kestose.

### 6.2. Increasing the Number of F. prausnitzii in the Intestine of Infants Treated with 1-Kestose

Allergies such as AD are dependent on T helper 2 (Th2)-derived immune responses characterized by the production of cytokines IL-4, IL-5, and IL-13, which promote the production of various mediators to induce allergic responses. Tregs are characterized by the production of IL-10, which suppresses the excessive activation of Th2 cells, thereby ameliorating allergic responses [20]. As *F. prausnitzii* is known to induce Treg differentiation and activation, it was thought that the 1-kestose-induced improvement in the AD symptoms might be correlated with an increase in the number of *F. prausnitzii*.

**Figure 4:**
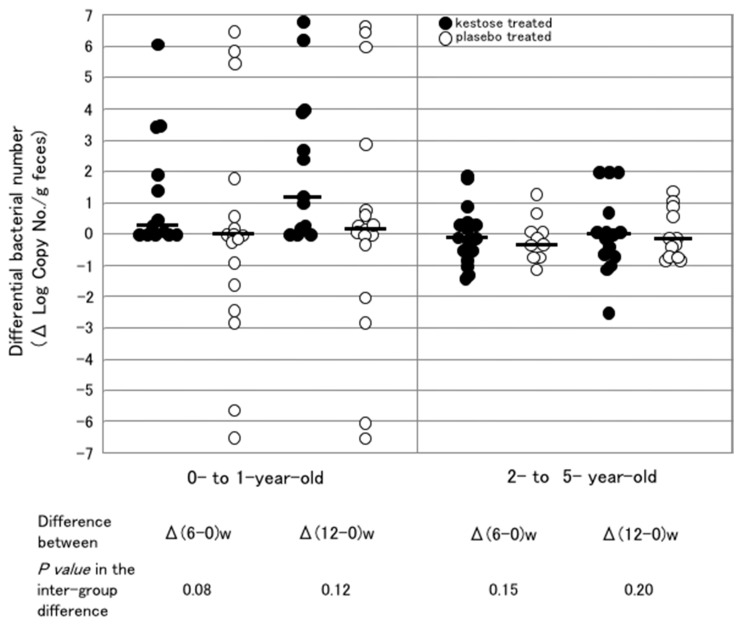
Changes in the number of *F. prausnitzii* after the treatment in the subgroups of younger and older infants. The left and right subpanels show the subgroups composed of subjects at 0 to 1 year of age and 2 to 5 years of age, respectively. Each symbol represents the differential change in the number of bacteria in subjects at 6 and 12 weeks after the start of the treatment. Filled and open circles indicate the subjects treated with 1-kestose and placebo, respectively. Bars represent the median of the differential bacterial numbers. (Unpublished figure).

Therefore, the count of *F. prausnitzii* was examined by real-time PCR in the feces of the infant patients with AD, who participated in a clinical trial to investigate the therapeutic effect of 1-kestose [21]. Regarding the colonization of *F. prausnitzii* in the intestine of those infants before treatment with 1-kestose, the bacterium count was almost undetectable level (<10^4^ log copy number/g feces) in 0- to 1-year-old infants but reached a level comparable to that in adults (10^7^ to 10^10^ log copy number/g feces) in 2- to 5-year-old infants. The infants were then allocated to the 1-kestose-treated group (*n* = 29) or placebo (maltose)-treated group (*n* = 29). The 1-kestose-treated group was orally given 1 g (<1 year old), 2 g (1 to 3 years old) or 3 g (4 to 5 years old) 1-kestose. After daily treatment for 12 weeks, fecal samples were collected to measure the bacterial count at 0, 6, and 12 weeks after the start of treatment (Figure 4). In all of the 1-kestose-treated subjects, the median number of *F. prausnitzii* at 12 weeks was about 10-fold higher than that at 0 week, and the increase was statistically significant between 12 and 0 weeks (data not shown). No such increase was observed in the placebo-treated group.

### 6.3. Difference in the Stimulating Effect of 1-Kestose on F. prausnitzii in Infantile Hosts with Different Ages

Next, to determine whether or not the stimulating effect of 1-kestose on *F. prausnitzii* was age-dependent, the change in the bacterial count was compared between two subgroups of 0- to 1-year-olds and 2- to 5-year-olds (Figure 4). In the 0- to 1-year-old subgroup, an exclusive increase (no decrease) in the differential count was observed in all subjects after 1-kestose treatment at 6 and 12 weeks, whereas both increases and decreases in the count were observed after placebo treatment (Figure 4, left subpanel). In the 2- to 5-year-olds subgroup, however, decreases as well as increases were observed in the 1-kestose treated groups at 6 and 12 weeks after treatment (Figure 4, right subpanel).

Precisely, why there was a marked difference in the efficiency of the 1-kestose-induced increase in the *F. prausnitzii* count between those two subgroups remained unclear. Hidaka et al. reported inter-individual difference in the bifidogenic effect exerted by FOS [22]. In their subjects who had a relatively small number of bifidobacteria, the bifidobacterial count increased up to 10,000 times the initial count after FOS treatment. In contrast, in the subjects who had a relatively large number of bifidobacteria, the bacterial counts did not show marked changes. Furthermore in our study, as mentioned in the previous chapter, the count of indigenous *F. prausnitzii* was very low in the younger subgroup in which a significant increase in the *F. prausnitzii* count was induced by 1-kestose treatment, however, it was already considerably high in the older subgroup in which no marked increase was induced by the treatment. These results imply that each group of bacteria (*Bifidobacterium* or *Faecalibacterium*) has its own inhabitable limit in the gut microbiota. Thus, the prebiotic effect on a bacterial group will be exerted only when inhabitable room remains for the group. However, given that different doses were used at the different age in our study, it may be impossible to rule out an effect of dose.

### 6.4. Effects of 1-Kestose on the F. prausnitzii Count in the Intestine of Adults

In order to examine the effect of 1-kestose on beneficial resident bacteria in adults, healthy adult volunteers were randomly allocated to either the placebo- or 1-kestose-treated group in our study. The placebo-treated group consisted of 6 subjects, and their median age and range were 39 years and 28–56 years, respectively. The 1-kestose-treated group consisted of 5 subjects, and their median age and range were 42 years and 27–60 years, respectively. The placebo- and 1-kestose-treated groups were orally given 5 g maltose (glucose-glucose) and 1-kestose daily, respectively, for 8 weeks. Fecal samples were collected to measure the bacterial count using quantitative real time PCR at 0 and 8 weeks after the start of the treatment (Table 1, unpublished data). In the 1-kestose-treated group, the number of *F. prausnitzii* after treatment was about 10-fold higher than that before treatment, and the increase was statistically significant (*p* < 0.05). The number of *Bifidobacerium spp*. was also significantly higher after 1-kestose treatment. However, no significant change in the count of *Clostridium cluster* XIVa or *Lactobacillus spp*. was found after treatment with 1-kestose. The placebo (maltose) treatment did not induce any significant change in the number of those bacteria examined in this study. These results clearly demonstrated a significant prebiotic effect of 1-kestose on *F. prausnitzii* in the gut of adults as well as infants.

## 7. Effects of 1-Kestose on the Butyrate Level

In humans, SCFAs, including butyrate, are predominantly generated in the colon and over 95% of them are thought to be rapidly absorbed [23], meaning that only a small proportion of SCFA are likely excreted in the feces. As such, human fecal samples may not accurately reflect the SCFA production in the colon; therefore, few studies actually describe the change in the level of butyrate in humans after treatment with prebiotics. Because of the difficulty of obtaining the colonic contents in humans, the butyrate concentration was also not measured in our human study investigating the effect of 1-kestose treatment using feces. Instead, we examined the effect of dietary 1-kestose on the concentration of butyrate in the contents of the cecum in rats [24]. The rats were divided into several groups (*n* = 8 per group) receiving a 0%, 2.5% or 5% 1-kestose diet for 4 weeks. The butyrate concentration was significantly higher in the 2.5% and 5% 1-kestose groups than in the 0% group. Moreover, the butyrate concentration level in the 5% 1-kestose group was approximately 10-fold higher than in the control group. And this group had neither diarrhea nor weight loss. Sakaguchi et al. [25] also reported an increased level of butyrate production after supplementation of prebiotic oligosaccharides in rats; Butyrate production increased approximately 5-fold and 2-fold by 10 % FOS and 10% GOS diets, respectively. Thus it is suggested that 1-kestose more efficiently activates *F. prausnitzii* to increase the production of butyrate as well as its number in the intestine of humans when compared with FOS or GOS, although the elevation of butyrate level by 1-kestose was confirmed in the rat system.

## 8. *F. prausnitzii*-Targeting Prebiotics

The concept of prebiotics was established more than 30 years ago. Thus far, FOS, inulin and GOS have been determined to fit the category of prebiotics, since their effects exclusively act through the enrichment of *Bifidobacterium* and *Lactobacillus* spp. Recently, the prebiotic concept has expanded mainly because of advances in research on indigenous microbiota. Consequently, a broader range of beneficial bacteria including butyrate-producing indigenous bacteria, such as *F. prausnitzii, Eubacterium rectale* or *Roseburia* spp., have been recognized and expected to be new probiotic strains [2]. Among them, resident *F. prausnitzii* in the intestine is predicted to be a good target of prebiotics, as has been described in this article.

Ramirez-Farias et al. [26] reported the effect of inulin on the number of *F. prausnitzii* in the gut. In their study, fecal samples obtained from healthy adults given 10 g/day inulin for 16 days showed a significant increase in the abundance of the bacterium compared with the baseline; however, the degree of increase was only about 50%. In contrast, in a study from our group (Table 1), the number of *F. prausnitzii* increased as much as 10-fold in adults given 5 g/day 1-kestose for 8 weeks. Dewulf et al. [27] performed a randomized controlled clinical trial using 30 adult women who were treated with inulin-type fructans (*n* = 15) or placebo (maltdextrin; *n* = 15) for 3 months (16 g/day). Treatment with inulin-type fructans led to a 2-fold increase in the abundance of *F. prausnitzii*. These clinical reports using inulin or inulin-type fructans showed that the prebiotic capacity to stimulate the growth of resident *F. prausnitzii* appeared far more efficient in the treatment with 1-kestose than with inulin or inulin-type fructans.

Furthermore, Scott et al. [7] examined the prebiotic substrates utilization patterns using 11 representative butyrate-producing bacterial strains. All 11 strains were able to grow on short-chain FOS, but this number decreased as the chain length of FOS increased. Inulin, which has a long chain length, was utilized by only 1 of the 11 strains. This report further suggested that 1-kestose, which has the smallest chain length among FOS, was able to exert a far more efficient prebiotic effect on *F. prausnitzii* than longer-chain FOS or inulin.

## 9. Future Prospects of 1-Kestose Concerning Clinical Application

Because 1-kestose was originally developed as an FOS component with an efficient bifidogenic effect, ordinary health endpoints targeted by FOS can also be used in assessment of 1-kestose. Constipation, bowel movement failure, and high blood lipid levels have been considered representative sicknesses to be treated or prevented with 1-kestose. Given that 1-kestose activates *F. prausnitzii*, the use of 1-kestose in Crohn’s diseases (CD) might be promising, as the treatment of CD with specific agents, such as Rifaximin [28] and Infliximab [29], has been shown to reverse the depletion of *F. prausnitzii*. The efficacy of 1-kestose for treating AD [19] has already been mentioned in this article. Moreover in this clinical trial, the 1-kestose treatment increased the number of *F. prausnitzii* in infants with AD. This result suggests the potential use of 1-kestose in infant formula. It is thought, at least, 1-kestose added to the formula will exert more bifidogenic effect than do FOS or GOS. However, it still needs to be elucidated whether an increase in the number of *F. prausnitzii* in infants is beneficial to their well-being if we try to administer 1-kestose to healthy infants for the increase of this bacterium. Improvements in insulin resistance with the 1-kestose treatment in obese rats has also been described by our group [24]. Taken together, these findings strongly suggest that 1-kestose has impressive potential as a new prebiotic targeting *F. prausnitzii*, a next-generation probiotic strain.

## Figures and Tables

**Table 1:** Effect of 1-kestose on the number of indigenous bacteria in the intestine.

Indigenous Bacteria Treated with ^a^		Log (No./g feces) of Bacteria	
		Before Treatment	After Treatment
*Bifidobacterium* spp.	Maltose	11.4 ^b^ (11.1–11.4) ^c^	11.3 (11.0–11.5)
	1-kestose	11.5 (11.0–11.6)	12.0 (11.8–12.1) *
*Clostridium cluster* XlVa	Maltose	11.9 (11.5–12.0)	12.1 (11.8–12.1)
	1-kestose	12.0 (11.8–12.1)	12.0 (12.0–12.3)
*F. prausnitzii*	Maltose	10.3 (10.0–10.8)	10.7 (9.1–11.2)
	1-kestose	10.4 (10.2–10.8)	11.1 (11.1–11.1) *
*Lactobacillus* spp.	Maltose	8.2 (7.9–9.0)	8.5 (7.9–8.6)
	1-kestose	8.8 (8.5–9.7)	10.8 (10.0–10.8)

**^a^**, Eleven adult volunteers were randomly allocated to maltase-treated group (*n* = 6) or 1-kestose-treated group (*n* = 5). Maltose- and 1-kestose-treated groups ingested 5 g of maltose and 1-kestose every day for 8 weeks. Before and after the treatment, the numbers of bacteria in the feces were enumerated by quantitative real–time PCR as reported previously (24). ^b^, Median; ^c^, Interquartile range; *, Significantly different (*p* < 0.05) between before and after treatment by Wilcoxon signed–rank test.
